# A Web-GIS tool for diagnosing spatial orientation of young adults: design and evaluation of Geo-Survey

**DOI:** 10.1038/s41598-023-45268-z

**Published:** 2023-10-30

**Authors:** Marcin Kulawiak, Dominik Krajnik, Marta Czaplicka, Agnieszka Dawidowicz

**Affiliations:** 1 Department of Geoinformatics, Faculty of Electronics, Telecommunication and Informatics, Gdansk University of Technology, Gdańsk, Poland; 2 Institute of Spatial Management and Geography, Faculty of Geoengineering, University of Warmia and Mazury in Olsztyn, Olsztyn, Poland

**Keywords:** Information technology, Environmental social sciences

## Abstract

Spatial orientation is the effectiveness with which one is able to assess the mutual location of objects relative to a point of reference or a system of coordinates. Traditionally, this ability has been evaluated through field navigation tests, which do not take into account the prevailing influence of free online maps and virtual walks on a person’s interpretation of space. In this context, this study presents a Web-GIS tool designed and developed to examine spatial orientation skills in the context of the used map type. The tool, named Geo-Survey, enables combination of survey questions with customized maps, providing users with a set of possible answer types. Moreover, using the unique concept of predefined answers, the tool attempts to automate the process of analysing research results. The tools’ performance is evaluated via assessing the spatial orientation skills of a group of young adults.

## Introduction

A map is the basic tool for visualizing the surface of the Earth on a two-dimensional plane with the use of graphic symbols, in a given scale, and according to strictly specified rules. Digital cartography has improved the accuracy of the calculations performed with the use of maps and made them widely available to the public through digital applications and platforms. A geographic information system (GIS) is one of such solutions which supports the creation, management, and analysis of spatial data. In the last decade, web-based GIS (also known as Web-GIS) have been used to develop various solutions, including geographic databases and data processing systems^1^, tools for geospatial data integration and analysis^2^, as well as applications for disseminating and visualizing large-volume datasets^3^. Due to the growing significance of spatial data in the modern world, GIS tools are frequently used in social dialogue to plan public spaces. This process gave rise to participatory geographic information systems (PGIS) which engage community members in planning the development of local landscape, urban systems and public communication routes, as well as sharing of spatial data and exchange of information between persons who have an interest in specific locations and resources^4^. Public participation geographic information systems (PPGIS), a form of PGIS, include SoftGIS tools which are online surveys for collecting information about the spatial location of objects, as well as data pertaining to the respondents' experiences and daily behaviours in space^5^. This combination of tools gave rise to geo-questionnaires (also known as geosurveys)^6–8^ which are the focus of the presented research.

A review of the literature indicates that geosurvey tools for diagnosing the spatial orientation skills of various age groups, in particular youths and university students, who have been most affected by rapid technological progress, have not been researched extensively to date. Recently, considerable advances in information technology (IT), the popularization of navigation tools based on global navigation satellite systems (GNSS) such as the global positioning system (GPS), GALILEO, or BeiDou, as well as rapid urbanization have significantly influenced our spatial orientation skills. The rapid growth of cities^9^ has necessitated the use of digital tools for navigating urban jungles^10^ and learning about urban space^11^. In consequence, people become familiar with geographic space not only by exploring it and memorizing images, but mainly through the use of public map websites and virtual walks. People who rely on digital maps, automated navigation tools, and GNSS to find their location become prone to losing the ability to read maps and navigate between places without external assistance. The above can significantly compromise their spatial orientation skills, in particular in the context of understanding the surrounding environment and the location of objects in the vicinity. The problem has become significant enough to warrant research into software tools for practice and development of spatial orientation in persons of all ages. In the case of children, this is done through entertainment software such as Pokemon GO^12^ or dedicated treasure hunting games^13^. Software directed towards teenagers and adults also uses computer vision and mobile applications, but takes a more direct form of applications dedicated to augmented reality (AR)^14^ and virtual reality (VR)^15^ spatial orientation training as well as multiple-aspect routing and navigation^16^.

Since the affected age groups are well versed in the use of modern technologies such as web-based software, it is a logical conclusion to employ an innovative geomatic tool in the form of a geosurvey to research their characteristics. For this purpose, a dedicated lightweight client–server application has been developed. The aim of the created Geo-Survey software was to provide quick and simple means of conducting geo-questionnaires on various topics, including spatial orientation. In this context, the application needed to provide several unique capabilities, including the ability to customize the used background map types as well as the ability to automatically analyse the validity of a respondent’s answers. The application was tested by performing a proof-of-concept survey of the spatial orientation skills of a group of university students.

In the above context, the presented research comprised a theoretical part (literature review), a conceptual part (development of measurement criteria), a design part (development of a geosurvey system), and a test part (a geosurvey involving an international group of computer science students).

## Background and motivation

**Figure 1:**
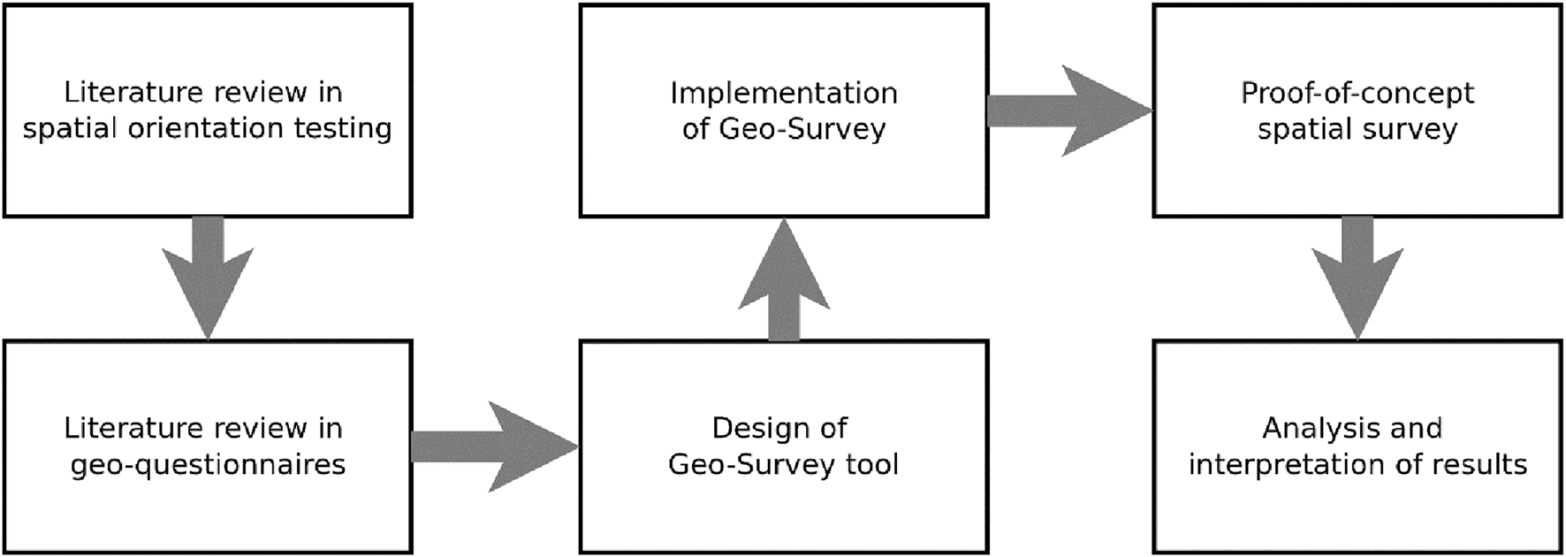
Methodology of the presented research. Source: own elaboration.

This study was motivated by the results of research into the assessment of spatial orientation in young adults which indicated that such studies could be carried out more efficiently with the use of a digital geo-questionnaire. The subsequent research into the available tools revealed severe limitations in areas such as map customization, which meant that a study comparing the influence of map type and contents on a person’s navigational skills would not be possible without creating a custom solution. This led to the development of our own geo-questionnaire tool, called Geo-Survey. Because the creation of the tool was motivated inter alia by the desire to test the influence of map type and content on a person’s ability to navigate it, the developed tool has been field tested during a proof-of-concept survey which aimed to investigate the extent in which using an unlabelled orthophotomap versus a partially labelled topographic map influences the spatial orientation of young adults who have been exposed to digital navigation systems for a prolonged period of time. All stages of the research process have been presented in Fig. 1.

This section contains the results of the literature reviews, while results of practical research are presented in subsequent sections.

### Assessment of spatial orientation

In subject literature, spatial orientation is defined as a set of abilities which enable a person to identify his or her position or the position of an object relative to a point of reference or a system of coordinates^17–20^ based on perceptions of distance, size and shape, as well as the mutual location of objects and the interactions between objects^21^.

Because spatial orientation is a process that involves numerous cognitive functions, diverse methods are needed to analyse an individual's spatial cognition skills^18^. In most studies, spatial orientation is assessed with the use of field navigation tests (wayfinding or map reading)^22^, as well as paper or digital tests. This is due to the fact that the process of reading maps requires understanding and interpretation of spatial information such as directions, distances, relationships between objects and terrain topography. This activates brain areas responsible for spatial memory and spatial imagination^23^. The latter, in particular, is the ability to create mental representations of spaces and objects in the mind, which allows one to understand and navigate the world around them^24^. Research by Downs and Stea^25^ has shown that spatial imagination can be improved by longer and more intense exposure to map reading. In turn, having a strong spatial imagination can benefit spatial orientation, spatial problem solving, and the ability to navigate the environment in general^26^. Hence, by testing the respondent’s map reading skill, one is also indirectly assessing their spatial orientation ability. However, geosurveys and similar tools have been rarely used in research on spatial orientation skills^27–33^.

The spatial orientation skills of university students have thus far been investigated to a varied extent and with the use of various methods, including through the construction of cognitive maps, by tracking the respondents' movement to a given destination, or by assessing their ability to read paper maps^32,34,35^. In particular,^29^ evaluated university students' knowledge about their immediate environment. According to the cited authors, geographical awareness and spatial memory are essential for navigating, exploring, and using space. As a result, geographical skills enable people to correctly interpret space and solve space-related problems^36^, especially since many problems in the modern world have an increasingly geographical character^37^.

### The influence of map type on spatial orientation

The human brain’s innate space interpretation mechanism, based on shape, colour and relative placement of detailed objects, has been refined by thousands of years of evolution. Traditional paper maps have only ever served as an extension of this mechanism. This being said, currently smartphones equipped with a GNSS module have become the main source of geographic information and a crucial spatial orientation tool for young people, to the extent where youths are becoming excessively reliant on them^29,38^. Navigation with a traditional map required maintaining a constant contact with the surrounding area, however smartphone navigation only reduces human interaction to following the path displayed on the smartphone screen. Several studies conducted over the last two decades have shown that prolonged exposure to digital navigation services may impair the natural process of acquiring spatial knowledge during travel^39–43^. Moreover, according to Acedo et al.^44^, human emotions and behaviours associated with a geographical area (sense of place) define (to a certain extent) the way in which people understand space, in particular urban space. When interaction with this space is limited, a person’s understanding of this space may also change.

Since virtually all smartphone navigation applications use abstract vector maps, it would be interesting to verify whether people raised in a digital world would be more used to navigating maps depicting semi-abstract representations of real-world objects (as seen on Google Maps or OpenStreetMap) than those built from photographs (and thus depicting space in a more traditional way). Logically, brain functions developed over thousands of years of evolution should still prevail over skills obtained during a relatively short (in comparison) period of upbringing. In this context, if the opposite could be identified in young adults who are known to be well acquainted with modern technologies, it could be indicative of future trends.

### State-of-the art in spatial questionnaires

A questionnaire which, in addition to standard questions that are found in traditional surveys, contains questions that are answered by placing points, lines, or polygons on an interactive map is often referred to as a geosurvey^6,45^. In this approach, the respondents' answers are set in a wider geographical context^46^ and can be analysed by spatial clustering or classification into groups based on respondent characteristics. By answering questions and performing tasks on an interactive map, the respondents become familiar with the tool, learn to read map symbols, localize objects in space, and improve their spatial orientation skills. Interactive features enhance the educational process and provide additional stimuli for the brain during learning. The use of information and communication technologies (ICT) is widely encouraged in geographical education^47,48^. GIS tools are also used in education to promote the development of spatial orientation skills, mainly through the use and creation of digital maps that expand the students' knowledge about the natural environment^49–51^.

Geosurveys may also be viewed as a type of computer-assisted web interview (CAWI) tools^52^, which are more effective in stimulating spatial cognition processes due to their integration with mapping tools^53^. In comparison to PPGIS tools, such as argumentation maps, geosurveys are completed independently by anonymous individuals who do not interact during the process^45^. A review of the literature on systems and platforms that support geosurveys indicates that this tool is used in five main areas: reporting problems, monitoring risks, social dialogue, social localization, and education. In the first area, a geosurvey can be modified to enable users to report accidents, system failures, or other problems^54–57^. In such cases, users do not answer direct questions, but report incidents and indicate their location on a map. Two types of applications can be used for this purpose:

1. Users exchange information about problems, indicate the exact location of these events on a map, and the resulting information is disseminated and delivered in real time;
2. Users report problems directly to the responsible authorities, such as the city hall or a public transport operator.

These applications differ from typical geosurvey platforms, however they engage community members enough to step beyond the traditional GIS framework.

Participatory GIS, including geosurveys, are increasingly used in public consultations as tools that promote debate about citizens' needs and preferences concerning local projects. The main disadvantage of the traditional model of public participation, where community members attend a meeting in a specific location (such as the city hall), is that it limits the spatial representation (e.g. by discouraging people who live on city outskirts from participation) and extent of the community members who can attend (e.g. by excluding elderly persons or people with disabilities who may have problems with reaching the meeting venue)^53,58,59^. A digital geosurvey can be used to collect information from respondents who usually do not participate in such initiatives^6^. Moreover, according to Czepkiewicz et al.^45^, geosurveys are popular among younger and better educated respondents who are familiar with digital tools. Geosurveys elicit information about the diverse needs and preferences of citizens, social groups, and civic societies, and they actively engage citizens in the process of planning solutions to local problems. As a result, geosurveys contribute to sustainable development, in particular by minimizing conflict in spatial planning^8,46,60–70^, assessing the risk of natural disasters^71,72^, evaluating ecosystem services in agroforestry landscapes^73,74^, evaluating access to health services^75,76^, assembling environmental psychology data^44^, assessing travel preferences^77^, and evaluating landscapes^78^.

In the work of Bąkowska et al.^79^, geosurvey tools were praised by most respondents who participated in public consultations. According to the respondents, geosurveys are widely accessible, offer numerous functionalities, engage community members, in particular youths, in local affairs, promote equal representation of the genders in social dialogue, and enable persons with disabilities to participate in consultations. Most critical opinions concerned technical problems and the risk of fraud because online voters can cast multiple ballots. The digital divide^66^, namely the potential exclusion of social groups with limited access to (and/or knowledge of) digital technologies was also identified as a problem. The latter issue was significant enough to justify simultaneous use of traditional consultation methods.

Solutions that offer similar functionalities or represent examples of good practices in PGIS include GeoCitizen^80^, American OpenTreeMap^81^, Polish LOPI^82^ (previously known as Geoankieta^83^), Finnish Kerrokartalla (Tell-on-a-Map)^84^, Maptionnaire^85^, Canadian Infill Planner^86^, and British FixMyStreet^87^. The latter platform in particular has many counterparts in other countries, including NaprawmyTo^54^, Zgłoś.Gdańsk^55^, and Zgłoś.24.pl^56^ in Poland; Street Guards and DAWAR^57^ in Egypt; and Pocitove Mapy (Emotional Maps)^88^ in Czechia.

All of the above applications feature a map where specific problems can be located with the use of markers, as well as a graphical user interface (GUI) which clearly describes available actions. Most of the analysed applications enable users to create accounts which permit them to comment on the information stored in the platform's database. The functionalities offered by these applications were compared to determine their suitability for conducting the planned survey (Table 1). The following features were analysed: availability of the source code, types of available questions, possibility to create predefined answers and automated analysis of survey results. Due to the specificity of the planned research, the main emphasis was placed on the type of answers which can be placed on the map (points, lines, polygons), the possibilities of configuring background maps, and the option of creating predefined answers to automate the analysis of the results in the summary panel.

**Table 1:** A comparison of applications for creating geosurveys based on selected features.

Application	Purpose	Open source	Custom questions	Type of questions	Type of answers on the map	Size of respondent group	Base map	Predefined answers
LOPI/Geoankieta	Geosurvey/geoforum	Yes	Yes	Marking a location on the map, descriptions, sliders	Placing points and polygons	Large	Choice of several predefined maps	No
Kerrokartalla	Geoforum	No	Not applicable	Marking a location on the map, descriptions	Drawing arbitrary shapes	Large	Choice of several predefined maps	No
Infill planner	Geosurvey	No	No	Marking a location on the map	Choosing from available areas	Small	Choice of several predefined maps	No
NaprawimyTo	Reporting problems	No	Not applicable	Not applicable	Placing points	Large	One predefined map	Not applicable
GeoCitizen	Geoforum	No	Yes	Project-related questions	Placing points	Large	One predefined map	No
OpenTreeMap	Data collection	Yes	Not applicable	Not applicable	Placing points	Large	One predefined map	Not applicable
Google forms + Google maps	Geosurvey	No	Yes	Descriptions	Choosing from available points	Small	Choice of several predefined maps	No

*Source*: own elaboration.

The results of the comparison (Table 1) point to a clear gap on the market of geosurvey systems. Simple open-source solutions are in short supply, and most applications are developed by companies or government institutions for a specific purpose or are a part of a larger (usually paid) platform. In the context of the planned study, the option of customizing the base map and creating predefined answers to automate the process of result analysis were the main limitations of the compared tools. None of the analysed applications offered the above functionalities, and the available open-source tools were either created for a different purpose (Open Tree Map) or exhibited an excessive architectural complexity which significantly limited their potential for adoption for the purpose of the planned research (LOPI). In view of these limitations, a dedicated tool was designed for the needs of this study.

## The developed Geo-Survey tool

Due to the limitations of the available solutions, a custom application, called Geo-Survey, has been developed for the purpose of the presented research. The tool has been designed with the use of modern IT technologies according to state-of-the-art in design of geosurvey applications. Basing on literature review and analysis of existing applications, the software was designed to deliver the following functionalities:

1. A map enabling the respondents to mark their answers with the use of points, lines, and polygons;
2. An option of formulating questions with predefined answers that are invisible to the respondents;
3. An option of exporting the respondents' answers for further processing and statistical analysis;
4. Integration of a system for comparing user responses with predefined answers, enabling automated analysis of survey results;
5. Support for desktop systems as well as touch screen devices, enabling application for research as well as educational purposes;
6. Intuitive GUI driven by responsiveness and simplicity.

### Architecture

**Figure 2:**
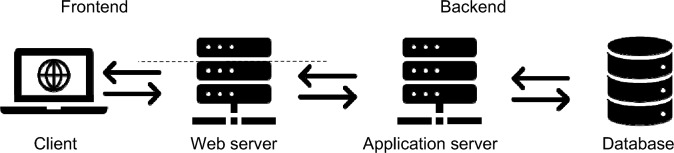
The architecture of the developed application. Source: own elaboration.

In order to enable further development and easy adaptation to the needs of similar studies, the architecture of the designed application had to be kept reasonably simple. To achieve this goal, the application was divided into two main elements (Fig. 2):

1. The backend part which reads stored geosurveys, stores user responses in the database, and calculates answer correctness during analysis of results;
2. The frontend part which presents data to the user and supports their interactions with the map.

In order to further simplify the application architecture, the database is stored in the cloud. This approach reduces the number of dependencies required to deploy the application, and it increases the application's security and reliability.

**Figure 3:**
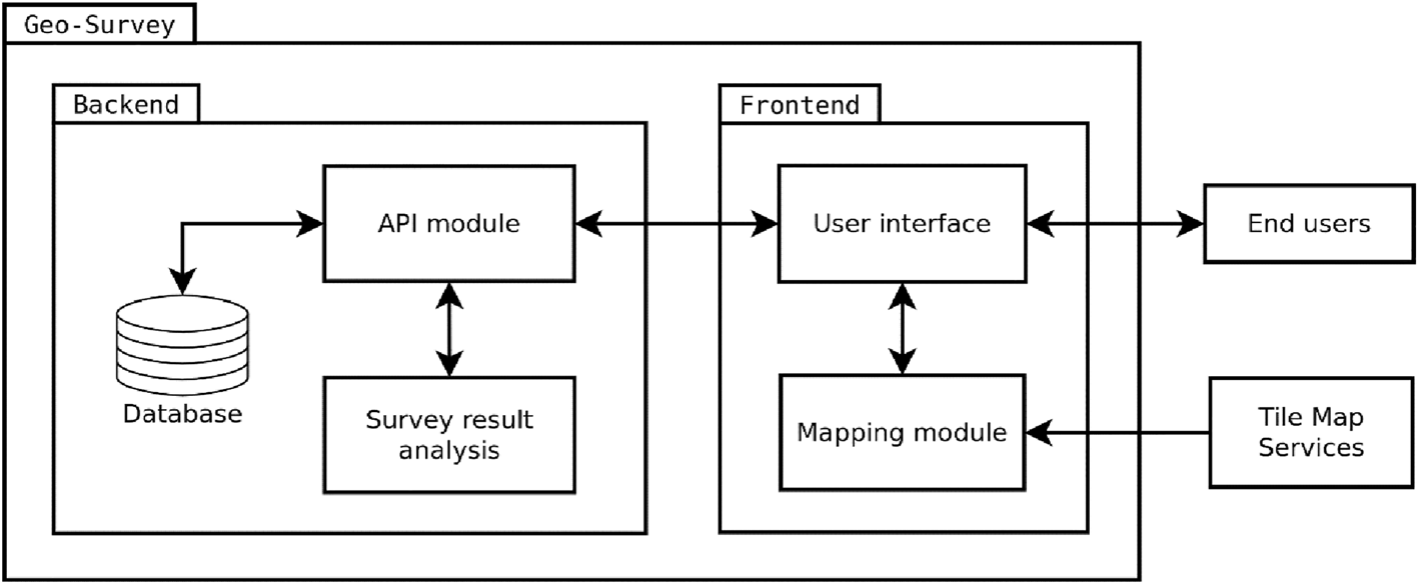
Diagram of system modules. Source: own elaboration.

Both elements of the system have been divided into modules, and each module has been implemented with the use of appropriate technologies. A diagram of system modules has been presented in Fig. 3.

The application was written in TypeScript, a statically typed language developed by Microsoft based on JavaScript, to which TypeScript code is compiled^89^. The frontend part of the application was developed with the use of the React.js library which facilitates the development of user interfaces^90^. Due to the sole use of DHTML, the application can be used in any modern web browser. On the backend side, the server was created with the use of Node.js, which is an open-source, cross-platform JavaScript runtime environment built on the V8 engine^91^. The application was built around an API module which supports user queries, provides access to surveys, stores answers and provides communication with the survey result analysis module. The database element has been realized with the MongoDB non-relational document database, which was selected due to its simplicity and adherence to the requirements of the designed application. In particular, the structure of geosurvey documents (which are the only element stored in the database) corresponded very well to the MongoDB BSON (binary JSON) data model. In the developed system, geosurvey documents comprise of questions and predefined answers encoded in the JSON format. These documents are stored on the application server alongside multimedia such as images attached to questions. Spatial data such as geosurvey answers is stored in the GeoJSON format, which is a standard for encoding various geographic data structures^92^.

The user interface for communicating with respondents and displaying the content downloaded from the server was developed in React.js with the use of the Material UI component library. Because the map module did not require sophisticated functionality, it was implemented with the use of Leaflet.js, which is a library for designing simple and effective mapping solutions. Because the library supports direct display of GeoJSON documents, geographic data placed in the system do not need to be additionally processed. The map module displays interactive maps and collects information about user interactions with the map. The map is selected by the administrator from the available tile map sources that are compatible with open standards for georeferenced map images such as the Web Map Service (WMS) or the Tile Map Service (TMS).

### Functionality

#### Survey design

**Figure 4:**
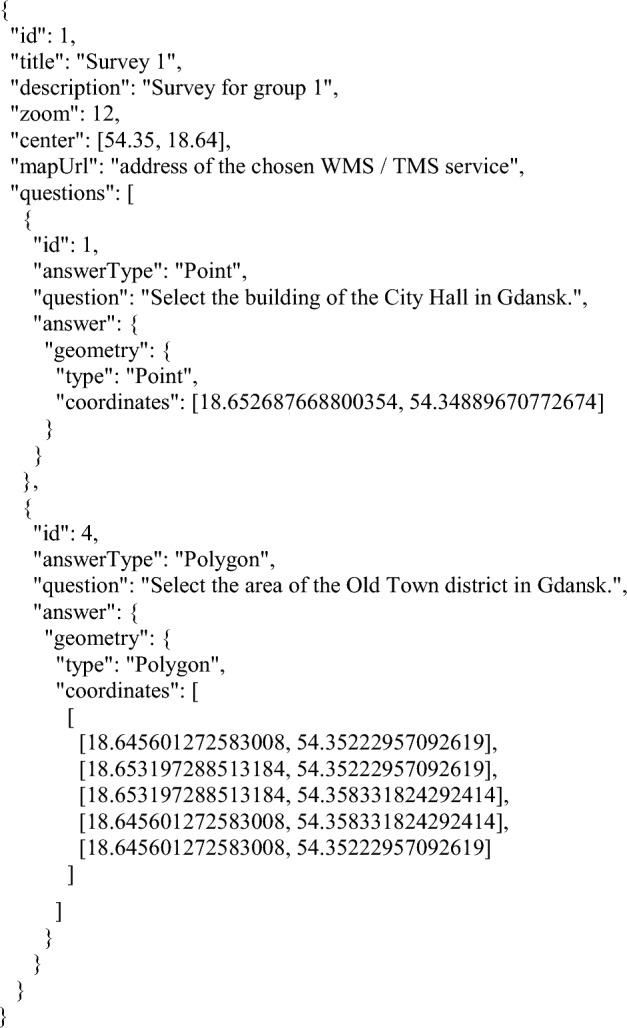
A fragment of a sample JSON file containing a geosurvey comprised of two questions with predefined answers. Source: own elaboration.

Geosurveys are added to the application by the administrator by placing a JSON file with predefined questions in an appropriate directory on the application server. An example of a JSON file containing a geosurvey comprised of two questions with predefined answers is presented in Fig. 4, with field descriptions available in Table 2.

**Table 2:** Description of fields used in the geosurvey JSON file.

Field	Description
id	Unique geosurvey identifier
title	Name of the survey
description	Description of the survey
zoom	Map zoom level at the beginning of the geosurvey
centre	Coordinates at the centre of the map at the beginning of the geosurvey
mapUrl	Link to the external WMS/ TMS service which provides a base map for the survey
questions:	Table of geosurvey questions
id	Unique question ID in a given geosurvey
answerType	Type of expected answer. The available values are: Point, LineString, and Polygon
question	Content of displayed question
answer	Predefined answer in the form of a GeoJSON geometry node
img	Optional path to an image displayed in the question

*Source*: own elaboration.

#### User registration

**Figure 5:**
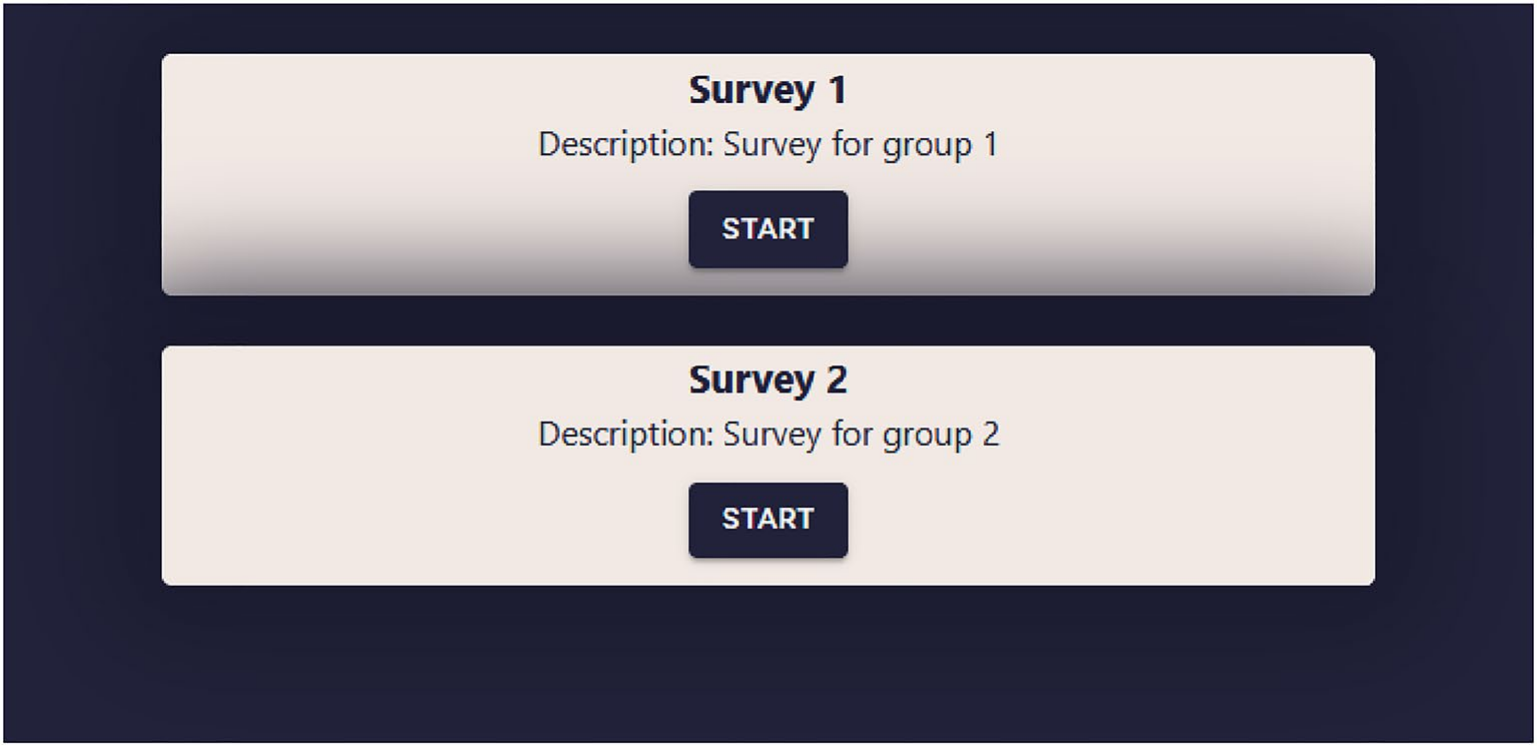
List of available geosurveys. Source: own elaboration.

Geosurvey documents encoded in this format are listed at application startup and displayed at the user's request. Clicking on an item from this list launches the selected geosurvey. The list of geosurveys created for the purpose of the presented study is shown in Fig. 5.

**Figure 6:**
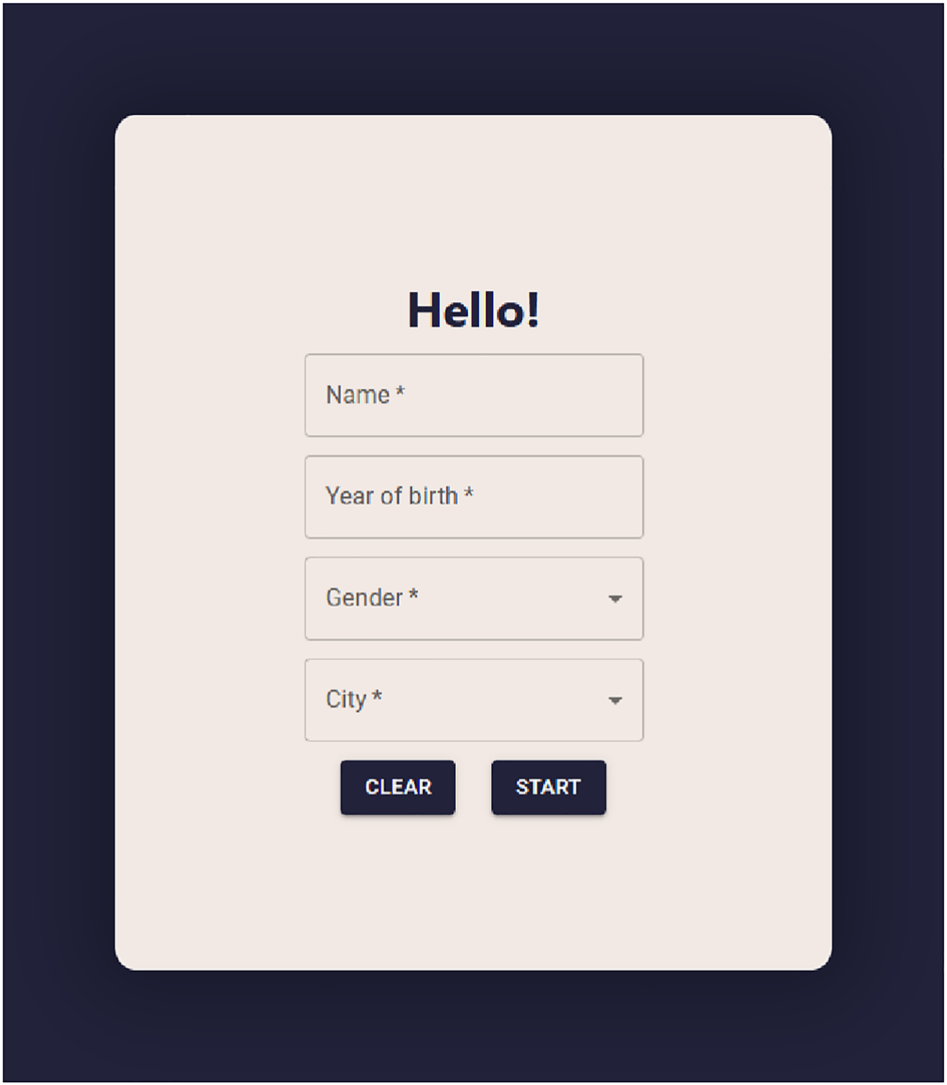
User profile questions shown in the initial screen of a geosurvey. Source: own elaboration.

The first screen that is displayed when a user launches a geosurvey contains several questions about respondent demographics, including age, gender, and place of residence. The collected data are used to profile users and analyse their responses. The surveyed person can enter their real name in the identifier field or use an alias to maintain anonymity. This initial geosurvey screen is presented in Fig. 6.

#### Answering questions

**Figure 7:**
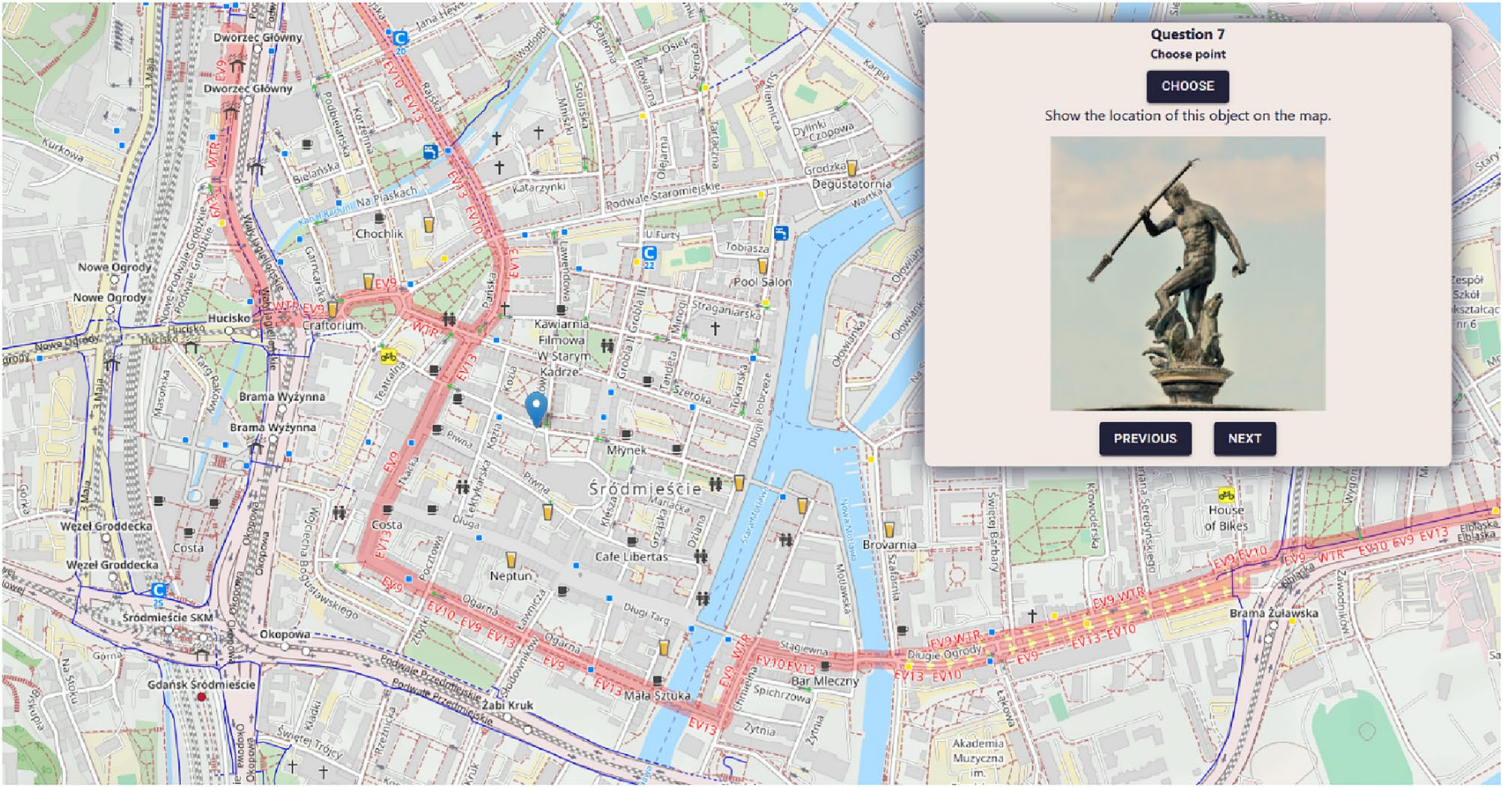
Geo-Survey displaying a question containing an image. Source: own elaboration using Open Cycle Map data (copyright OpenStreetMap.org contributors, licensed under the Open Data Commons Open Database License (ODbL)).

After completing their demographic profile in the first screen, the user can proceed to answer geosurvey questions. Each geosurvey contains a predefined number of questions, and the user can proceed to the next question only when the current question has been answered. The given answers are buffered locally, giving users the ability to navigate between answered questions and modify their responses. Geosurvey questions may also contain images, as shown in Fig. 7.

**Figure 8:**
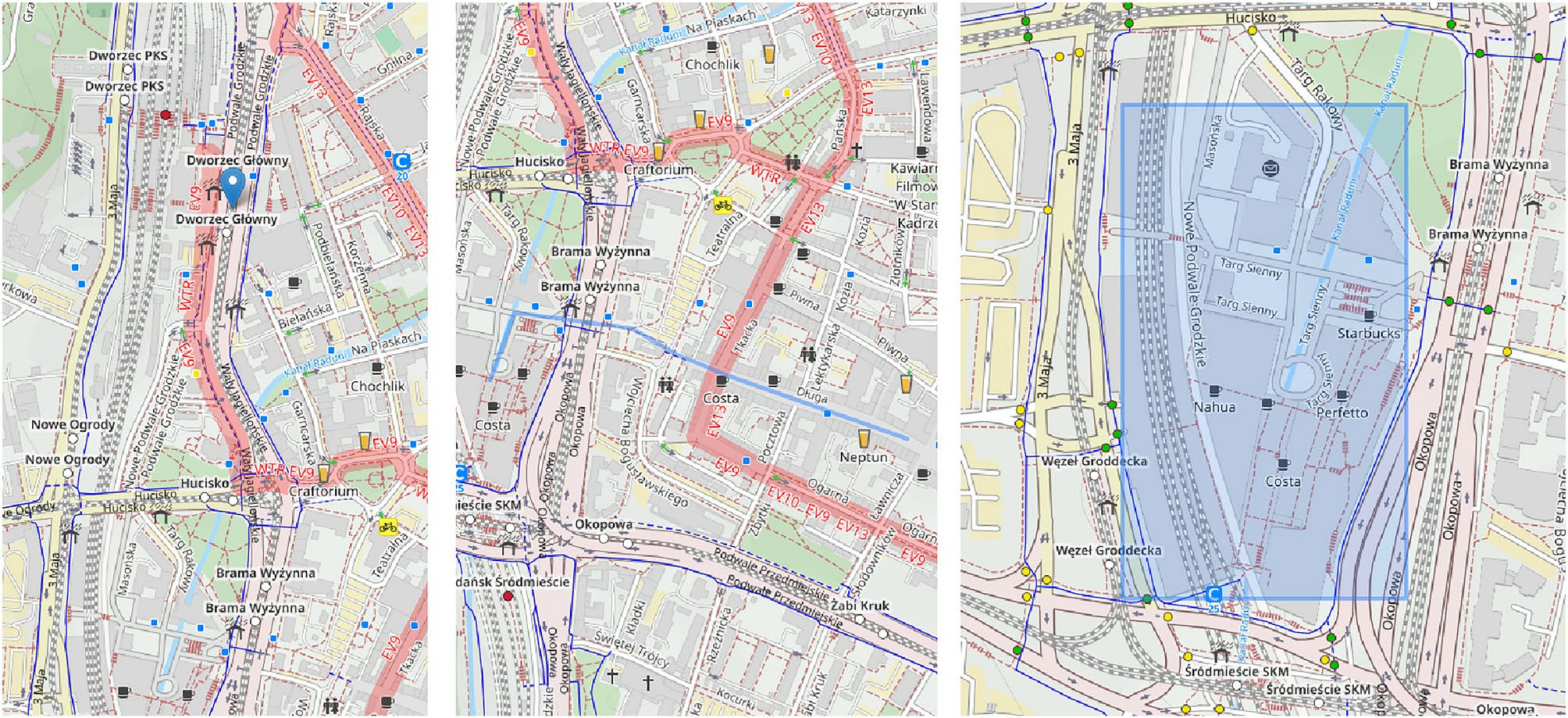
Possible types of answers in Geo-Survey: point (left), line (centre) and polygon (right). Source: own elaboration using Open Cycle Map data (copyright OpenStreetMap.org contributors, licensed under the Open Data Commons Open Database License (ODbL)).

The expected type of answer is defined by the author of the geosurvey. Depending on the question, the respondent is expected to mark a geographic area or feature by drawing a point, polyline or rectangle. Different types of answers are presented in Fig. 8.

**Figure 9:**
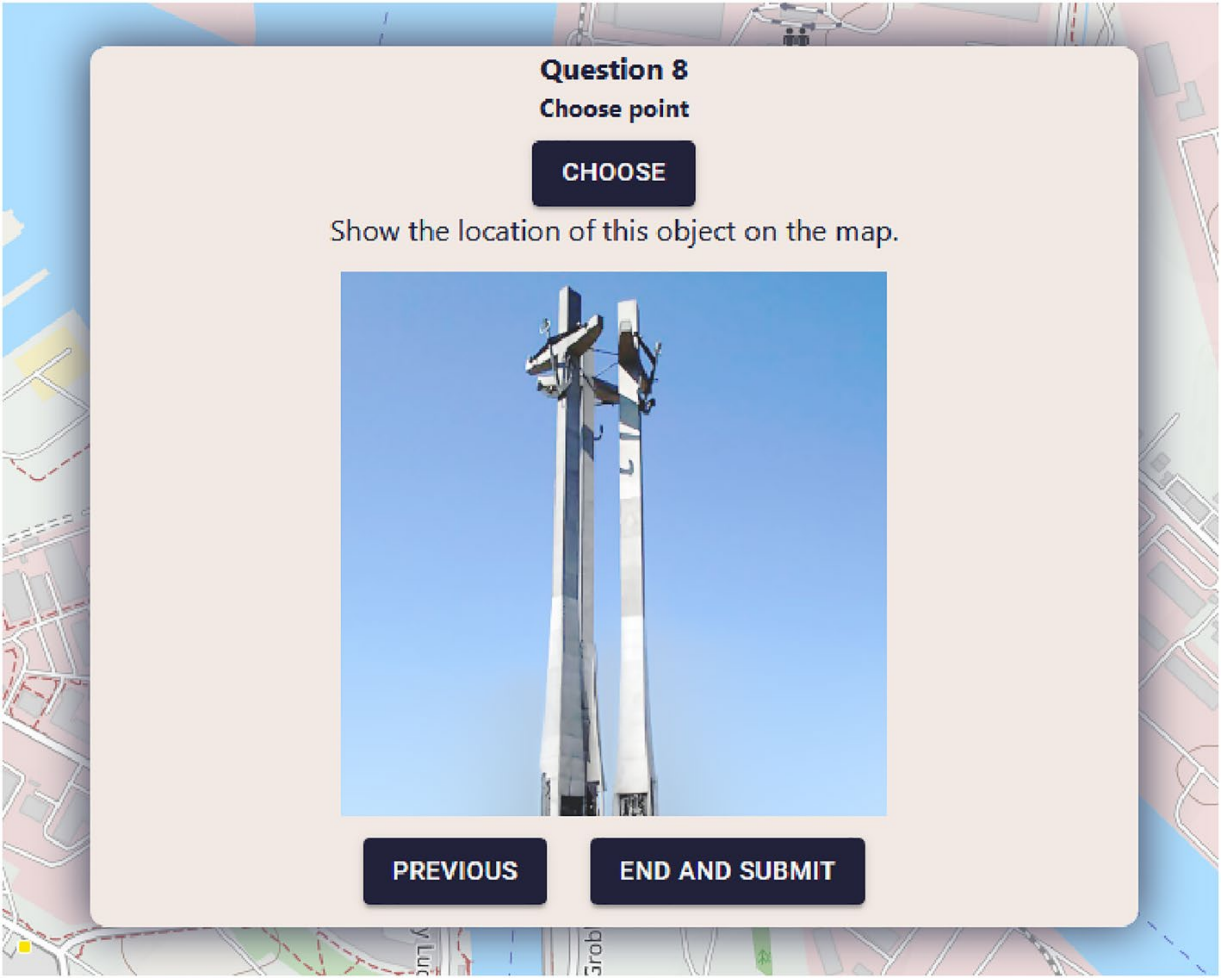
View of the last question with the option of ending the geosurvey. Source: own elaboration using Open Cycle Map data (copyright OpenStreetMap.org contributors, licensed under the Open Data Commons Open Database License (ODbL)).

To save their responses, the user has to answer all questions and click on “End and submit” (Fig. 9). When the answers have been submitted, the application returns to the initial screen and awaits the next user.

#### Analysing responses

**Figure 10:**
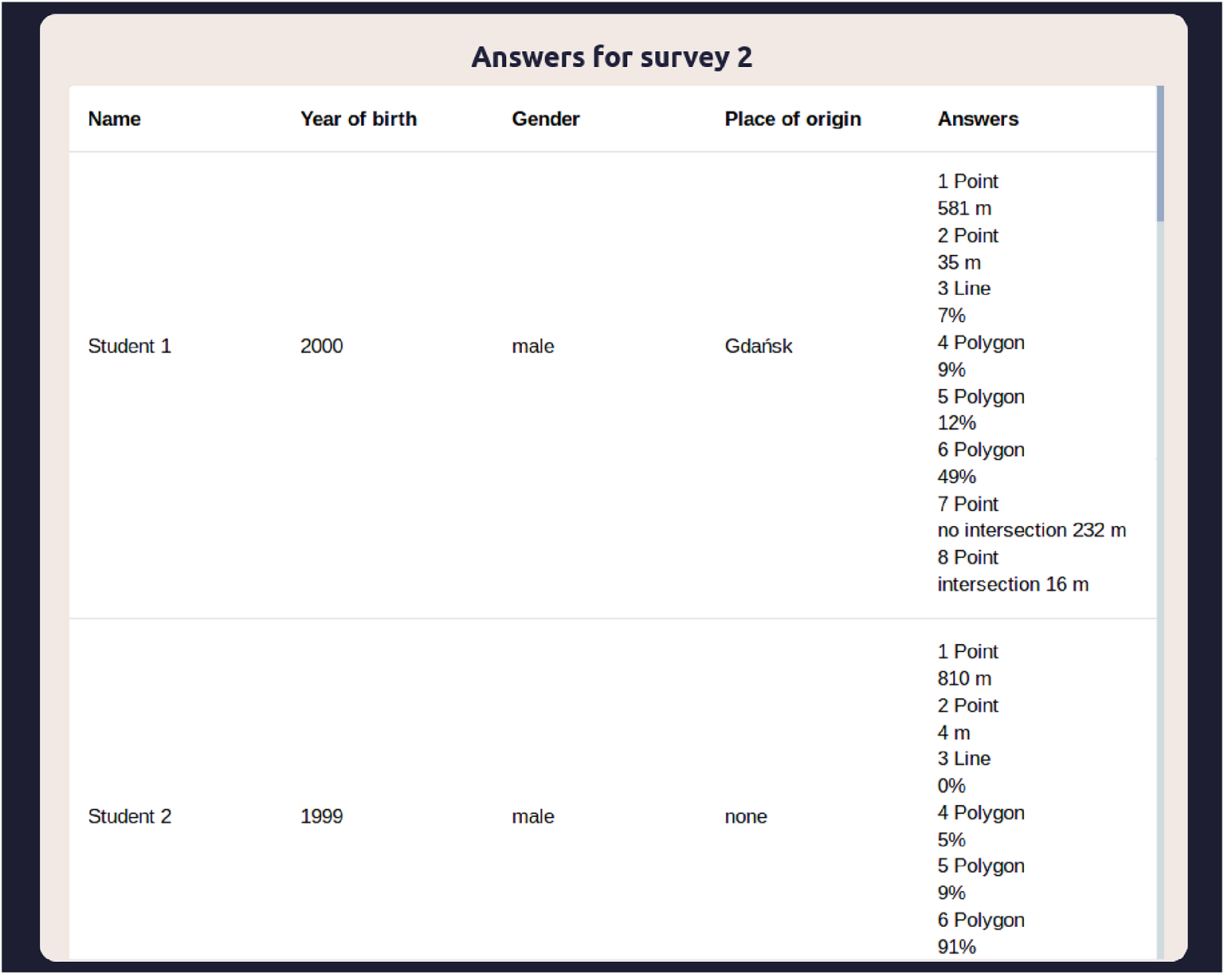
Sample view of geosurvey results. Source: own elaboration.

The system’s automated result analysis module enables the administrator to access the statistics calculated for every respondent at any time. A sample list of automatically calculated geosurvey responses is presented in Fig. 10. The type of question and the predefined answer influence the method of calculating the results. In point-type questions, the predefined answer can be either a point or a polygon. In the former case, the result is calculated as the distance between the point placed by the respondent and the predefined answer. In the latter case, the result is a true/false value which indicates whether the response falls within the area of a predefined polygon, accompanied by the distance between the given response and the centre of the predefined polygon. In line-type questions the predefined answer can only be a polygon, and the result is calculated by calculating the length of the line segment drawn by the respondent which falls into the predefined polygon, and comparing it to the total length of the line as well as and size of the polygon. In polygon-type questions the only available type of predefined answer is a polygon, and the accuracy of the given answer is calculated in a two-step process. The first step involves calculating the areas occupied by the predefined polygon, the respondent’s polygon, and the polygon representing the intersection between these polygons. The next step involves calculating a minimum from the quotient of the area of the intersection and the predefined answer and the quotient of the area of the intersection and the respondent's answer. The resulting minimum value, expressed as percentage, represents the extent to which the given answer overlaps with the predefined answer. Survey results can also be exported in JSON format, in which case the results are not calculated automatically, and the responses are presented as geographic data in GeoJSON format. This enables the answers to be easily imported e.g. to standard GIS software packages for further processing.

## Sample application and test of Geo-Survey

### Survey design and respondent profiles

In May 2022 the developed application was used to survey the students of the Gdansk University of Technology Faculty of Electronics, Telecommunications and Informatics in Gdansk, northern Poland. Twenty-three students enrolled in the sixth semester of an international computer science program participated in the study. The surveyed group consisted of 16 persons identifying as male and 7 persons identifying as female. All respondents were born between 1999 and 2001 and came from several European Union Member States. The aim of the survey was to test the influence of the employed map type on the students’ ability to locate key objects in the city of Gdansk.

Every participant completed a total of three questionnaires: the initial survey, the main geosurvey, and the post-survey. The initial survey aimed to assess the respondents' potential knowledge of Gdansk through the following questions:

1. How would you rate your knowledge of Gdansk (possible answers: high, medium, low);
2. Were you born within a distance of 30 km from Gdansk (possible answers: yes, no);
3. Have you been living within a distance of up to 30 km from Gdansk in the last 3 years (possible answers: yes, no).

Since knowledge about the surrounding environment is determined directly by a person’s experiences^45,67,93^ and the distance from their place of residence, the given answers were used to divide the respondents into two groups with similar self-reported spatial awareness of the city. Both groups were also balanced in terms of representation, with the first group consisting of 8 males and 3 females, and the second group consisting of 8 males and 4 females.

**Figure 11:**
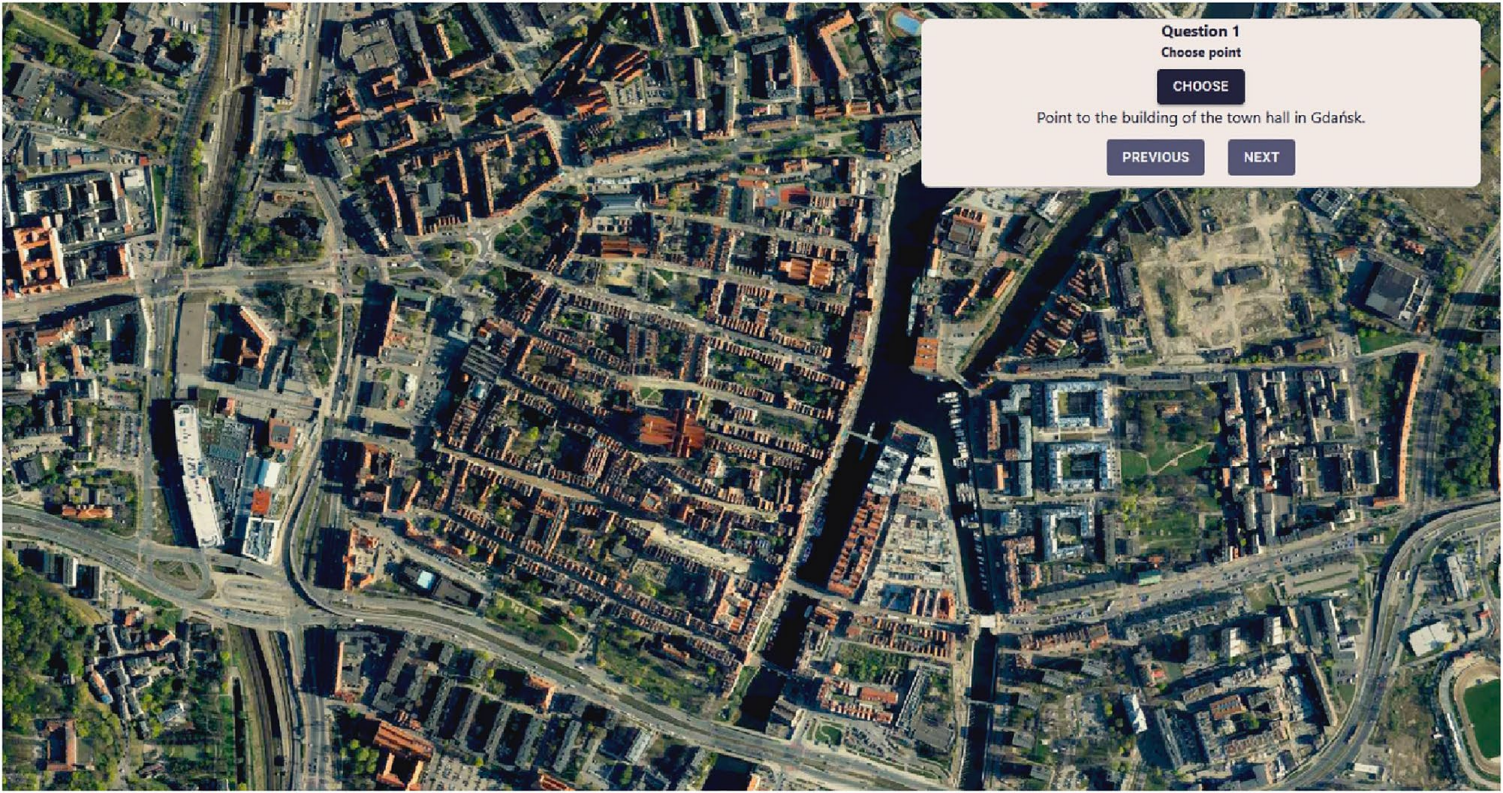
Geo-Survey questionnaire based on an orthophotomap, which was used for group 1. Source: own elaboration using Geoportal data (licensed under the INSPIRE *No Conditions to Access and Use* License).

**Figure 12:**
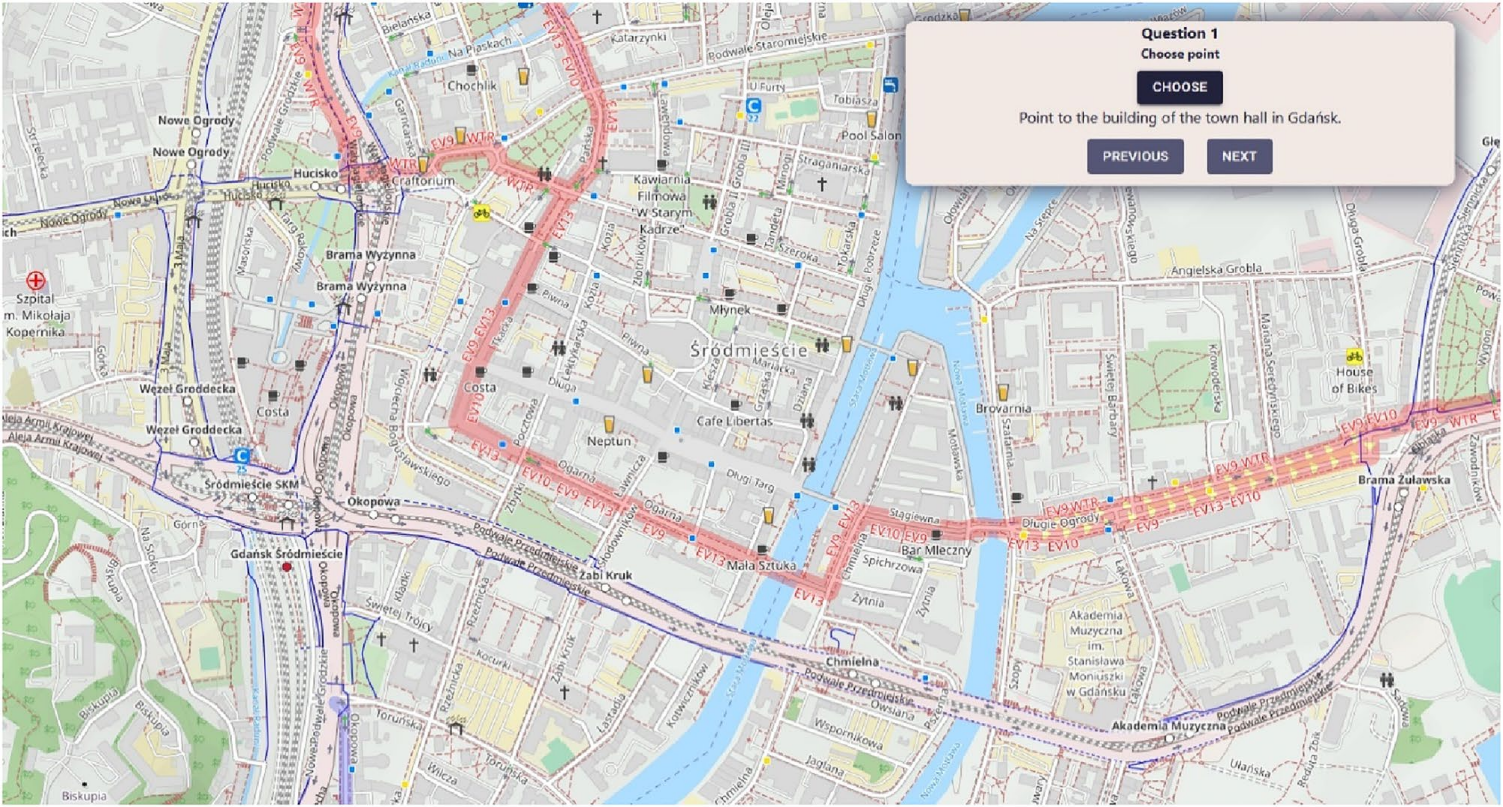
Geo-Survey questionnaire based on Open Cycle Map, used for group 2. Source: own elaboration using Open Cycle Map data (copyright OpenStreetMap.org contributors, licensed under the Open Data Commons Open Database License (ODbL)).

The survey took place in a computer laboratory, with every participant sitting in front of a PC workstation. Both groups took the survey in their separate turns, with all group members completing the survey at the same time. All students have previously completed a course in Geographic Information Systems, and thus were familiar with both types of maps used in the study. Each group had to answer the same eight questions, with the only difference between them being the type of given base map. The first group was presented with an unlabelled orthophotomap, whereas the second group received Open Cycle Map, which is a variant of Open Street Map devoid of many landmark labels. The reasoning behind these choices was to investigate the differences in respondents’ ability to interpret maps on the basis of visual indicators only (in the case of the unlabelled orthophotomap) in comparison to relying on topographic terrain layout and street names, but without markers indicating the placement of the features they were requested to locate (in the case of Open Cycle Map). The differences between these types of reference maps are presented in Figs. 11 and 12.

The geo-questionnaire for each group contained the same list of questions which is presented in Table 3. To answer these questions, the respondents had to draw a point, a line, or a polygon on the map. All questions concerned downtown Gdansk, which is the most popular part of the city, to increase the probability that the respondents would be familiar with the searched objects. The questions have been categorized into three difficulty classes. Finding the location of the railway station, Neptune fountain and town hall was considered “easy” (because the town hall and the fountain are the city’s hallmarks, while the train station may be easily found by following the train tracks), locating the monument of fallen shipyard workers was considered of “medium” difficulty (due to it being a lesser hallmark) and the identifying the old town and main town districts was considered “hard” (because the historical centre with Neptune’s fountain and town hall is located in the main town district, while the old town district is located further to the north and houses less known attractions such as the monument of fallen shipyard workers and the train station).

**Table 3:** Questions and types of answers in the geosurvey.

No	Question	Type of answer	Type of predefined answer	Question rating	Notes
1	Indicate the location of the Gdansk Town Hall building	Point	Point	Easy	The Gdansk Town Hall is one of the city’s hallmarks; its location should be well known even to tourists
2	Indicate the location of the main railway station in Gdansk	Point	Point	Easy	The building of Gdansk main railway station is a well-known monument, however it is located away from city centre and may not be familiar to all visitors
3	Mark the shortest distance between the Town Hall building and the Gdansk Forum	Line	Polygon	Medium	The Gdansk Forum is a popular commercial complex located in a straight 500 m line from the City Hall. Its location should be easy to establish for anyone aware of the City Hall’s location
4	Indicate the area of the Old Town district in Gdansk	Polygon	Polygon	Hard	The Old Town district lies to the north of Gdansk historical centre. This question is meant to gauge how advanced is the respondent’s knowledge of the city
5	Indicate the area of the Main Town district in Gdansk	Polygon	Polygon	Hard	The Main Town district houses the city’s historical centre and most of its tourist attractions. This question is meant to assess if the respondents can differentiate it from the Old Town district
6	Indicate the area of the Gdansk Forum shopping centre	Polygon	Polygon	Easy	The Gdansk Forum complex occupies a trapezoid area delimited by four of the city’s largest streets. It should be easy to mark on the map
7	Mark the location of this object on the map (image of Neptune's fountain)	Point	Polygon	Easy	The Neptune fountain is one of the city’s hallmarks; its location opposite the Town Hall should be well known even to tourists
8	Mark the location of this object on the map (image of the Monument to the Fallen Shipyard Workers, Trzech Krzyzy Square)	Point	Polygon	Medium	The monument pays respects to shipyard workers who were killed during protests in December 1970. It is located in the city’s Solidarity square, near a historical shipyard gate, away from the city centre

*Source*: own elaboration.

There was a time limit of 20 min on completing the survey, in order to give the participants enough time familiarize themselves with the given map type but at the same time discourage them from spending too much time on trying to answer any of the given questions. As a result, since neither the town hall, the Neptune’s fountain, the fallen shipyard workers monument nor the main and old town districts were labelled on either map, their location could not have been easily found without at least a basic knowledge about their whereabouts.

After completing the geo-questionnaire, the respondents were asked to participate in a brief post-survey to summarize their experiences. The aim of the post-survey was to determine which elements of the application are consistent with expectations, and which should be improved. The post-survey contained the following questions:

1. How would you rate your overall experience with the app?
2. Were the questions clear?
3. Is the user interface clear and easy to navigate?
4. Did you get lost at any point in the survey?
5. How would you rank the graphic design?

The results of all three surveys are presented in the following sections.

## Results and discussion

### Results of initial survey

As previously mentioned, the purpose of the initial survey was to divide the respondents into two groups with similar levels of knowledge of Gdansk. The results of the survey and the resulting division of respondents into groups are presented in Table 4 for group 1 (orthophotomap) and in Table 5 for group 2 (OSM). The user ID’s given in Tables 4 and 5 are consistent with those used in the analysis of geosurvey answers presented in the following sections.

**Table 4:** Results of the initial survey for group 1.

ID	Born within a distance of < 30 km from Gdansk	Has been living within a distance of < 30 km from Gdansk in the last > 3 years	Knowledge of the city
1	No	No	Low
2	No	No	Medium
3	Yes	Yes	High
4	Yes	Yes	High
5	No	Yes	Low
6	No	No	Low
7	No	No	Medium
8	Yes	Yes	Medium
9	No	No	Medium
10	No	No	Low
11	No	No	Low

*Source*: own elaboration.

**Table 5:** Results of the initial survey for group 2.

ID	Born within a distance of < 30 km from Gdansk	Has been living within a distance of < 30 km from Gdansk in the last > 3 years	Knowledge of the city
1	Yes	Yes	High
2	Yes	Yes	Medium
3	No	No	Medium
4	No	No	Medium
5	No	No	Low
6	No	Yes	Medium
7	Yes	Yes	Medium
8	Yes	Yes	High
9	No	Yes	Low
10	No	No	Medium
11	No	Yes	Low
12	No	No	Low

*Source*: own elaboration.

As indicated in Table 4, 27% of group 1 respondents were born in Gdansk or the surrounding areas, and 45% had been living in the vicinity of Gdansk for more than 3 years. Only 18% of the participants rated their knowledge of the city as “high”, whereas 36% rated it as “medium”, and 46%—as “low”.

In group 2, 33% of the respondents were born in Gdansk or the surrounding areas, and 58% had been living in the vicinity of Gdansk for more than 3 years. Despite the above, only 16% of the participants rated their knowledge of the city as “high”, whereas 33% rated it as “medium”, and 51%—as “low”.

### Results of the geo-questionnaire

**Figure 13:**
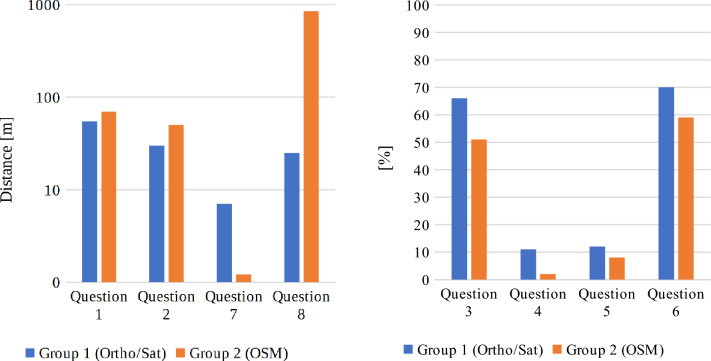
Median difference from correct answers in terms of distance (left, less is better) and percentage (right, more is better). Source: own elaboration.

**Figure 14:**
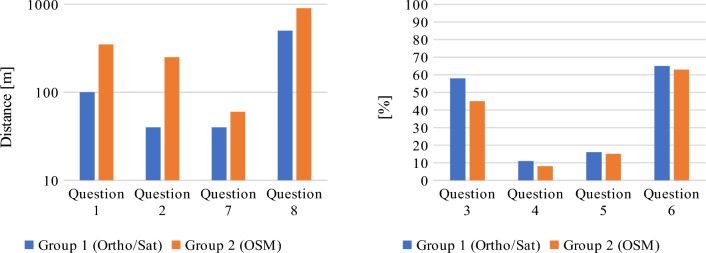
Mean difference from correct answers in terms of distance (left, less is better) and percentage (right, more is better). Source: own elaboration.

The results of the geo-questionnaire are presented in Figs. 13 and 14. Questions 1, 2, 7 and 8 were answered by placing a point marker on the map, and the accuracy of these answers was determined by calculating the distance between the provided answer and the predefined answer. In questions 3, 4, 5 and 6, the participants had to draw a line or a polygon, and therefore the accuracy of these answers was determined by calculating the percentage in which they matched the predefined answers.

**Figure 15:**
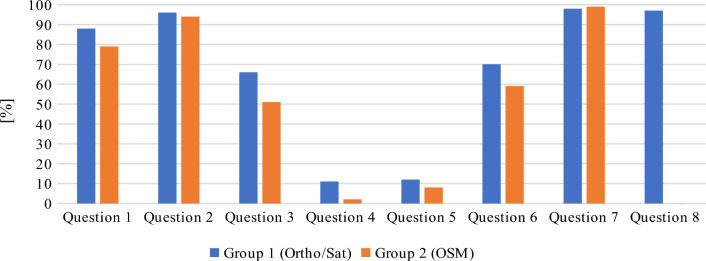
Normalized median of geosurvey results (more is better). Source: own elaboration.

**Figure 16:**
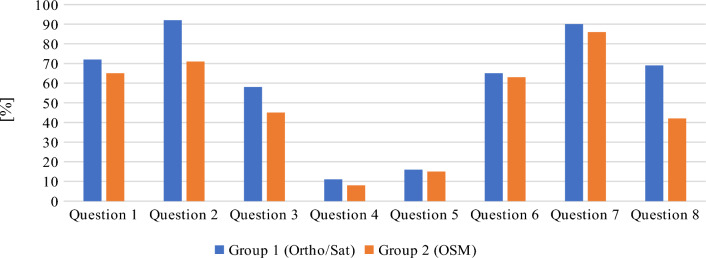
Normalized mean of geosurvey results (more is better). Source: own elaboration.

The presented figures display mean and median differences from correct answers, calculated by the system directly from the given geosurvey responses. However, because using two different metrics would constitute sub-optimal means of overall data investigation, the distance results were normalized to percentage values for the needs of further analyses. For this purpose, the maximum difference between the answer given by the respondent and the predefined answer was set at 250 m. Considering the initial scale of the given base map and the average distances between features in question, it was decided that distances above this threshold indicated that the respondent was not familiar with the location of the given object. The distance interval of 0–250 m was rescaled to 0–100%, where the distance of 0 m represented 100% accuracy, and the distance of 250 m or more denoted 0%. The normalized results are presented in Figs. 15 and 16. In consequence of normalization the results can now be presented on a single diagram, and the process also minimized the effect of “gross” errors on mean and median values. As a side effect, because most answers to question 8 by respondents from group 2 were much further off the mark than the chosen maximum distance, the median correctness percentage value for this group’s answers has become zero (see Fig. 15).

The presented data indicate that both groups of respondents could quite accurately locate Gdansk’s most famous landmarks, including Neptune's fountain, the Town Hall building and the main railway station. Interestingly, both groups managed to more precisely pinpoint the location of both the Neptune’s fountain (Question 7) and railway station (Question 2) in comparison to the Town Hall building (Question 1), which is located only 30 m from the Neptune’s fountain. The majority of respondents also properly identified the area of the Gdansk Forum shopping complex (Question 6). The shortest path from the Town Hall to Gdansk Forum (Question 3) is a straight line represented by the Dluga street, ending with an underground passage. The fact that the underground passage is not well indicated on either base map, alongside the prior requirements of knowing the locations of Town Hall and Gdansk Forum, is likely why the accuracy of replies to this question was a bit lower than to questions 7 and 1. The monument of fallen shipyard workers (Question 8) may be of international fame, but it is of less interest to general public, which (combined with its somewhat remote placement over a kilometer from the historical center) likely caused it to be among the most problematic objects to locate on the map. It is also the question in which the differences in the accuracy of given responses was the greatest between both groups. The distribution of the answers to question 8, ranked from the most to the least accurate, is presented in Table 6. As it can be seen, the respondents either gave the correct answer or marked a location that was more than 250 m away from target. This distribution pattern was not observed in any of the other questions. Moreover, some of the respondents who were not familiar with the location of the monument of fallen shipyard workers had been living in Gdansk for over 3 years. The above results could indicate that these respondents have poor spatial orientation skills, poor spatial memory, or are not highly skilled in using maps to locate themselves and other objects in space.

**Table 6:** Distribution of answers to question 8.

Group 1 (Ortho/Sat)	Score	Has lived in Gdansk for more than 3 years	Born in Gdansk	Group 2 (OSM)	Score	Has lived in Gdansk for more than 3 years	Born in Gdansk
Respondent 5	94.8	No	No	Respondent 2	96.8	Yes	Yes
Respondent 1	94.4	No	No	Respondent 8	96.4	Yes	Yes
Respondent 4	94.4	Yes	Yes	Respondent 7	96.0	Yes	Yes
Respondent 7	94.4	No	No	Respondent 10	96.0	No	No
Respondent 8	94.4	Yes	Yes	Respondent 1	93.6	Yes	Yes
Respondent 9	94.0	Yes	No	Respondent 3	0.0	No	No
Respondent 3	93.6	Yes	Yes	Respondent 4	0.0	No	No
Respondent 6	92.8	No	No	Respondent 5	0.0	Yes	No
Respondent 2	0.0	No	No	Respondent 6	0.0	Yes	No
Respondent 10	0.0	No	No	Respondent 9	0.0	Yes	No
Respondent 11	0.0	No	No	Respondent 11	0.0	Yes	No
				Respondent 12	0.0	No	No

*Source*: own elaboration.

**Figure 17:**
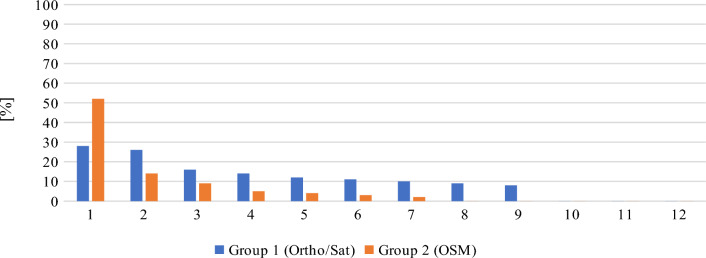
Distribution of answers to question 4 (more is better). Source: own elaboration.

**Figure 18:**
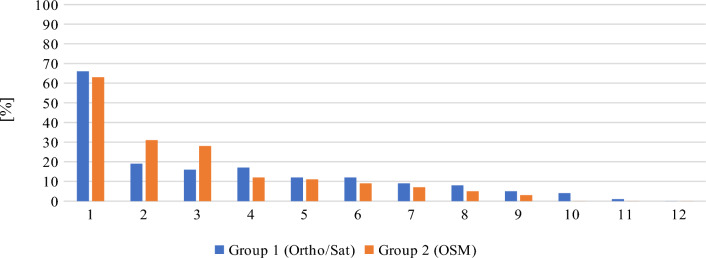
Distribution of answers to question 5 (more is better). Source: own elaboration.

As far as the general accuracy of given answers is concerned, the replies to question 4 (location of the Old Town district) and question 5 (location of the Main Town district) clearly deviate from the mean in both groups. This may be due to the fact that, unlike in most urban centers, the Old Town district in Gdansk does not feature the most valuable historical architecture and is not the main tourist attraction. Instead, the historical center is located in the Main Town District. In consequence, answering these questions correctly required excellent knowledge of the city, which could only realistically be expected from long-time residents. The distribution of the answers to questions 4 and 5, ranked from the most to the least accurate, are presented in Figs. 17 and 18, respectively. Out of the total number of 46 answers to questions 4 and 5, only 3 were characterized by an accuracy of over 50%. Interestingly, these answers were given by two persons (one from each group) and one of these persons only managed to locate the Main Town district.

As for the differences between groups, group 1 respondents generally had better success in locating objects on the map, which could be attributed to the fact that the orthophotomap was much easier to navigate despite the absence of any labels. Respondents using the orthophotomap had better access to information about environmental detail such as the accurate shapes and colours of buildings, trees, streets etc., which allowed them to more easily locate familiar objects and thus better orientate themselves on the digital map. The differences between the groups were not always significant, but they were noted in all questions. To verify the above results and minimize the effect of “gross” errors on mean scores, in Table 7 the average score of every respondent was compared with the average score in a given group.

**Table 7:** Geosurvey results—comparison of answers in both groups (more is better).

Group 1 (Ortho/Sat)			Group 2 (OSM)		
id	Knowledge of the city	Score (%)	id	Knowledge of the city	Score (%)
9	Medium	72	8	High	82
8	Medium	72	10	Medium	59
7	Medium	70	7	Medium	54
3	High	65	12	Low	51
5	Low	64	2	Medium	50
6	Low	59	4	Medium	47
1	Low	56	5	Low	43
10	Low	53	3	Medium	42
4	High	51	9	Low	37
2	Medium	45	11	Low	37
11	Low	16	1	High	33
			6	Medium	25
Average score		57	Average score		47
Average score excluding outliers		59	Average score excluding outliers		45

*Source*: own elaboration.

**Figure 19:**
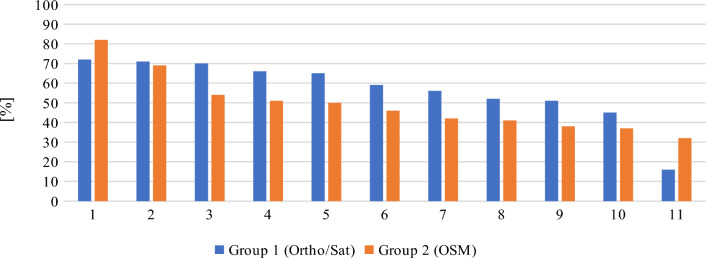
Distribution of the mean percentage of answer correctness for each respondent. Source: own elaboration.

As shown in Table 7, despite the fact that the studied population was relatively small, the elimination of outliers did not affect the average score, which indicates that the initial classification process was effective in creating two groups with similar levels of knowledge and spatial orientation skills, as well as sufficient differentiation. This can also be seen in the distribution of the mean percentage of answer correctness for each respondent, as presented in Fig. 19. The answers given by respondents in each group approximated the respective mean values, and group 1 respondents received noticeably higher average scores than group 2 participants. Regarding outliers, one respondent in group 1 received a score that was significantly below the average, whereas two respondents in group 2 received scores that were significantly above the average. These results also indicate that both groups were characterized by similar levels of knowledge and spatial orientation skill.

**Figure 20:**
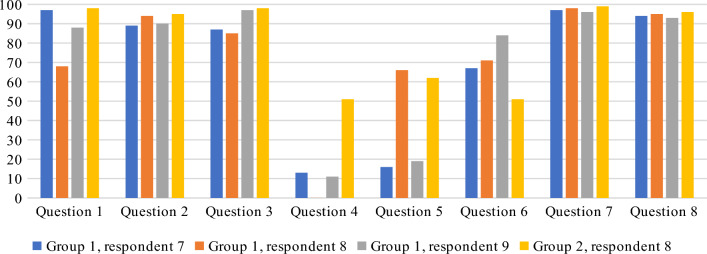
Distribution of the answers given by four highest-scoring respondents. Source: own elaboration.

In the surveyed population, four respondents achieved a mean percentage of answer correctness above 70%. Their responses are compared in Fig. 20. The results confirm that questions 4 and 5 were most challenging even for the highest-scoring respondents. Interestingly, despite the fact that questions 4 and 5 were complementary (respondents who were aware that the historical city center is located in the Main Town district should be also familiar with the general location of the Old Town), only one of the two respondents who located the Main Town district with more than 50% accuracy was familiar with the location of the Old Town district.

### Results of the post-survey

**Figure 21:**
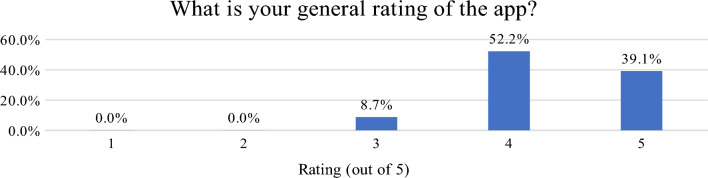
Rating of the overall experience with the Geo-Survey application. Source: own elaboration.

**Figure 22:**
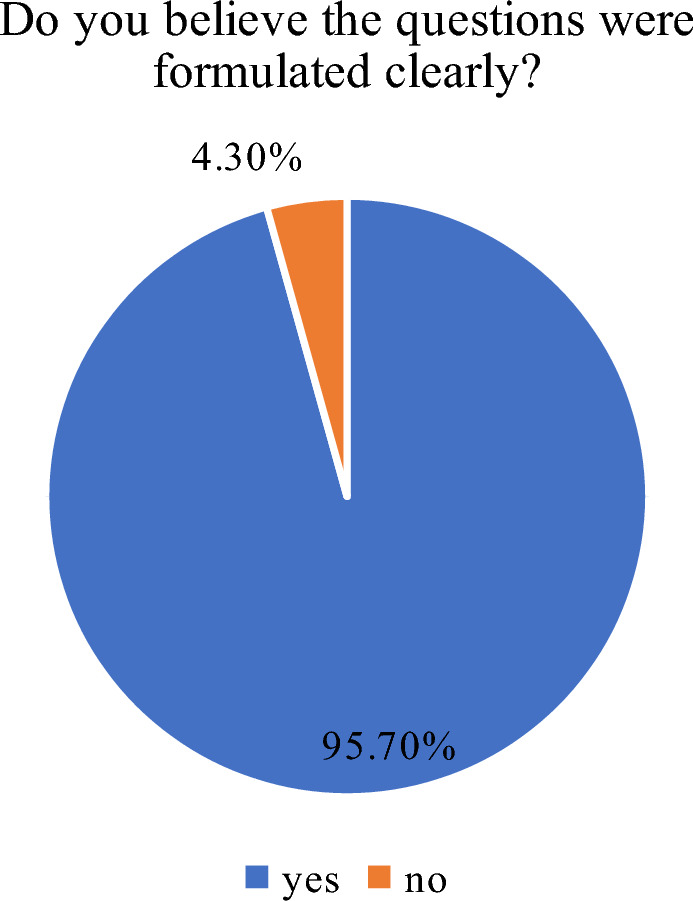
Rating of question clarity. Source: own elaboration.

The results of the post-survey are presented in Figs. 21 and 22. The application received positive feedback from all respondents, and most of them had no doubts regarding the questions contained in the geosurvey. Very few problems were reported, most of them were related to the participants' unfamiliarity with the searched objects, or the lack of detailed instructions for drawing lines on the map. In the latter case, the respondents misunderstood this functionality or became lost after making a mistake.

### Ethical approval

The authors have no conflicts of interest to declare that are relevant to the content of this article. The authors confirm that all methods were carried out in accordance with relevant guidelines and regulations. The authors state that all experimental protocols have been prepared in accordance to the guidelines of the Gdansk University of Technology Committee on Research Ethics. The study was approved by the University of Warmia and Mazury Committee on Research Ethics. Informed consent was obtained from all subjects.

## Conclusions

The results of the presented study have shown that it is not only possible to assess spatial orientation with a digital geo-questionnaire, but using such a tool can significantly enhance the research process. The developed Geo-Survey tool has shown to enable the assessment of respondents' spatial orientation skills and their ability to read and navigate cartographic materials through locating geographical features. The developed tool can be used in research studies that rely on modern technologies to explore problems associated with geographic education and spatial orientation. The application offers unique features unavailable in competitive solutions, including the ability to precisely configure base maps, as well as an automated data analysis module that facilitates statistical processing and ranking of results. The application was tested by performing a proof-of-concept survey which aimed to verify whether the type of used background map affected the spatial orientation skills of a group of IT students. The conducted geosurvey revealed that although the surveyed young adults were well versed in using technology to navigate urban systems and despite the fact that such navigation usually relies on labeled topographic maps, the respondents found it much easier to navigate an unlabeled orthophotomap than a detailed topographic map. The results indicate that by presenting real-world colors and shapes of spatial features, aerial images of the street network better facilitate the identification of specific objects. The study also demonstrated that the self-reported knowledge about the given area or the fact that the respondent resided in that area were not always correlated with their ability to find relevant places or objects on the map. The obtained results also suggest that the prolonged exposure to abstract representations of reality in combination with automated navigation on smartphone devices did not have a significant effect on the respondents’ innate navigation skills (as shown by their superior performance of reading an orthophotomap versus a more abstract topographic map), which would indicate that their spatial imagination (and, in turn, spatial orientation) have not been affected by the omnipresent technology. This data suggests that future research into spatial orientation may produce more insightful results should it be performed using orthophotomaps.

The results of the post-survey indicate that some system functionalities could be improved. According to the respondents, a short tutorial on how to use drawing tools would improve user experience. In the future, a geosurvey design module similar to the response module could be introduced to generate geosurveys with a user-friendly interface. This solution could enable the development of the Geo-Survey application into a web platform, enabling online design and conduction of geosurveys. This functionality could be implemented without increasing the architectural complexity of the application, which would mean that it would remain easily modifiable and adaptable to specific needs. Further research could also facilitate optimization of the approach to assessing spatial orientation skills on various scales and in different social groups.

## Data Availability

Data for all experiments are available from the corresponding author upon request.
